# Efficient Mixed-Type Wafer Defect Pattern Recognition Based on Light-Weight Neural Network

**DOI:** 10.3390/mi15070836

**Published:** 2024-06-27

**Authors:** Guangyuan Deng, Hongcheng Wang

**Affiliations:** 1 School of Electrical Engineering and Intelligentization, Dongguan University of Technology, Dongguan 523808, China; 221115194@dgut.edu.cn; 2 School of Computer Science and Technology, Dongguan University of Technology, Dongguan 523808, China

**Keywords:** wafer map, defect pattern recognition, light-weight neural network, attention mechanism, large kernel convolution

## Abstract

Wafer defect pattern recognition can help engineers improve the production process of semiconductor chips. In real industrial scenarios, the recognition of mixed-type wafer defects is difficult and the production scale of semiconductor wafers is large, which requires high accuracy and speed in wafer defect pattern recognition. This study proposes a light-weight neural network model to efficiently recognize mixed-type wafer defects. The proposed model is constructed via inverted residual convolution blocks with attention mechanisms and large kernel convolution downsampling layers. The inference speed of the inverted residual convolution block is fast, and the attention mechanism can enhance feature extraction capabilities. Large kernel convolutions help the network retain more important feature information during downsampling operations. The experimental results on the real Mixed-type WM38 dataset show that the proposed model achieves a recognition accuracy of 98.69% with only 1.01 M parameters. Compared with some popular high-performance models and light-weight models, our model has advantages in both recognition accuracy and inference speed. Finally, we deploy the model as a TensorRT engine, which significantly improves the inference speed of the model, enabling it to process more than 1300 wafer maps per second.

## 1. Introduction

### 1.1. Background

**Figure 1:**
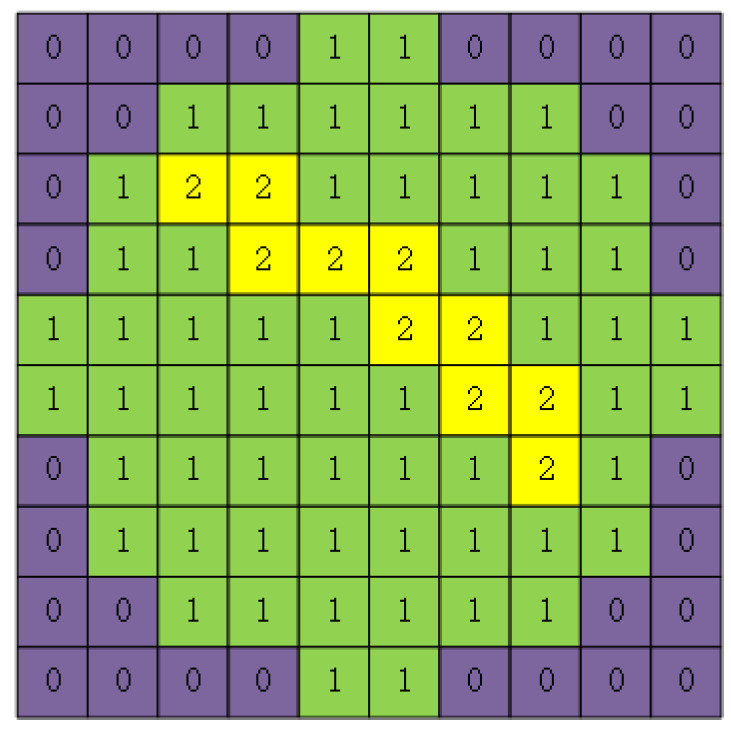
Raw wafer map with a Scratch defect.

Semiconductor wafers are silicon-based substrates used for fabricating integrated circuit chips. The fabrication of semiconductor integrated circuit chips can be divided into four steps: wafer fabrication, wafer test, assembly, and final test [1]. Wafer fabrication involves a series of complex processes, such as silicon ingot growth and extraction, cutting, grinding, polishing, photolithography, chemical etching, etc. Any problems arising in the processes will lead to wafer defects. Wafer test refers to the process of assessing the electrical functions of each die on wafers before slicing and packaging. In this process, a wafer is divided into multiple dies, and then the electrical function of each die is tested by using a wafer probe. The dies with normal electrical function will be labeled as qualified, and the test results of all dies on a wafer are saved on the same wafer map [2]. Wafers with abnormal electrical functions will form a certain shape pattern of defects on the wafer maps [3]. Semiconductor engineers determine whether wafers have defects by observing the wafer maps obtained from testing. Based on the type of defects, they infer the causes of the faults in the wafer fabrication process, aiming to improve the wafer production process and enhance product yield [4,5]. For example, hardening of polishing pads can result in Scratch defects [6], and a 10 × 10 raw wafer map with this defect tested by the wafer probe is shown in Figure 1. In the wafer map, 0 represents background, 1 represents normal dies, 2 represents defective dies, and the defective dies form a Scratch shape.

In recent years, the semiconductor industry has developed rapidly, and the production scale of integrated circuit chips has been increasing. Traditional methods of manually identifying wafer defects are no longer applicable. Because the manual method requires high labor costs and relies on the work experience of semiconductor engineers. Furthermore, various chemicals are used in the wafer manufacturing process, and long-term exposure to wafers may pose a threat to human health [7]. Currently, automated wafer defect recognition methods based on machine learning and deep learning have gradually become mainstream, and with the rapid development of deep learning technology, deep learning methods have taken a dominant position. Under the guidance of Moore’s Law, the precision of integrated circuit chips continues to increase, and the complexity of semiconductor wafer manufacturing also increases accordingly. This has led to an increase in the probability of mixed-wafer defects, making it more difficult to automatically detect wafer defects [8].

Many of the existing automated identification methods for wafer defects such as [9,10,11,12,13] do not consider mixed-type wafer defects, and there are also many studies, such as [11,14,15,16], that pursue recognition accuracy improvement but ignore the inference speed of models. In real industrial scenarios, there are mixed-type wafer defects, and the identification of such defects is challenging [17,18]. The production scale of real wafer factories is relatively large, so wafer defect recognition methods with slow inference speed are difficult to be applied in the actual wafer production process [19]. Thus, these existing methods cannot adapt well to the increasingly complex semiconductor wafer manufacturing processes and the growing production scales in real industrial scenarios.

Light-weight neural networks can effectively reduce the computational cost of deep learning models for wafer defect recognition because of their advantages of fewer parameters and low computational cost [20,21]. The definition of light-weight neural networks is generally relative. In my opinion, neural networks with a similar number of parameters and similar computational complexity to the well-known light-weight convolutional models, such as the MobileNet series and the ShuffleNet series, can be referred to as light-weight neural networks. This means that the number of parameters in a light-weight neural network is in the millions or even lower, and the number of floating-point operations is in the billions or even lower.

### 1.2. Contributions

This study proposes a light-weight neural network model based on inverted residual convolution blocks with attention mechanisms and large kernel convolution downsampling layers. In the proposed model, the CBAM (convolutional block attention module) is introduced to enhance feature extraction capabilities, a separate downsampling layer is designed to accelerate inference speed, and large kernel convolution is applied to reduce the loss of detailed features in downsampling operations. Finally, this study employs the stochastic depth technique to optimize model training and deploys the model as a TensorRT engine to improve inference speed. With these improvements, we have developed a high-accuracy and fast-inference automated model for mixed-type wafer defect recognition. We conducted comparative experiments on the open-source Mixed-type WM38 dataset with some existing high-performance models and light-weight models. The results show that the proposed model has advantages in both recognition accuracy and inference speed.

### 1.3. Structure of This Paper

The structure of this article is organized as follows: Section 1 is the introduction of this paper. Section 2 discusses some related work about wafer defect pattern recognition tasks. Section 3 provides a detailed introduction to the light-weight neural network model proposed in this study. Section 4 describes the experimental details and results. Section 5 presents the conclusion and provides suggestions for future work.

## 2. Related Work

### 2.1. Wafer Defects and Wafer Map Data

There are eight common types of wafer defects, including Random, local, Edge-Local, Center, Edge-Ring, Scratch, Near-Full, and Donut. Associated defects and causes are summarized as follows [6]:

- Random defects are caused by problems in the clean room environment, which result in dust particles and debris adhering to the surface of the silicon wafer;
- Local defects and Edge defects are caused by uneven grinding, polishing, cleaning, or non-uniform wafer surface;
- Center defects are caused by uneven polishing of the silicon wafer surface during chemical mechanical process;
- Edge-Ring defects are caused by uneven grinding of the silicon wafer, misalignment between layers during photolithography, or problems with chemical etching;
- Scratch defects are caused by mishandling, hardening of polishing pads or agglomeration of particles;
- Near-Full defects are caused by Agglomeration of multiple systematic and random defects;
- Donut defects are caused by non-uniform surface, equipment handling or hardening of polishing pads.

Mixed-type wafer defects refer to multiple different defects on a single wafer, which are caused by failures in multiple processes during wafer fabrication.

**Figure 2:**
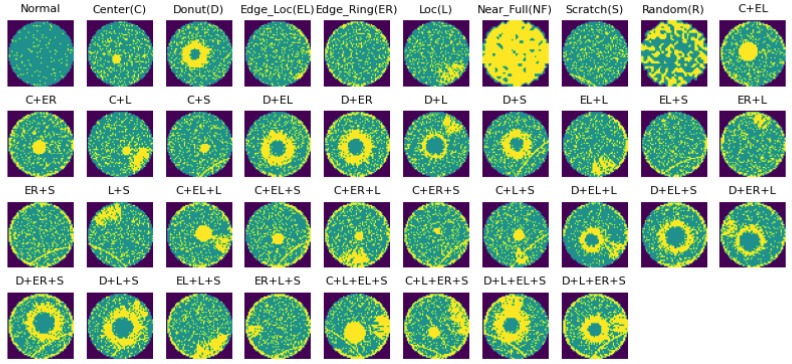
Various categories of Mixed-type WM38.

WM811K [22] is the open-source wafer dataset with the largest quantity of wafer map data. It contains 811,457 wafer maps, including the eight single-defect categories mentioned above and one defect-free category. However, there are no mixed-type defect wafer maps in WM811K. Mixed-type WM38 [14] is another open-source wafer dataset, which contains 38,015 wafer maps, including a category of defect free wafer maps, eight categories of single-defect wafer maps and twenty-nine categories of mixed-type defect wafer maps, as shown in Figure 2. In addition, the method of using Mixup to generate mixed-type defect wafer maps [17,23] is applied for wafer defect pattern recognition.

### 2.2. Wafer Defect Recognition Based on Machine Learning

Early machine-learning-based wafer defect recognition models were generally combined with some feature extraction methods. The two-stage SVM (support vector machine) model [22], which was trained with geometry- and radon-based features, achieved automatic wafer defect recognition. In this model, the first SVM determines whether there is a defect and the second SVM determines the type of defect. The voting ensemble classifier [24], which consists of four machine learning modes—logistic regression, random forests, gradient boosting machine, and artificial neural network—was applied for wafer defect recognition. And the density-, geometry-, and radon-based features were extracted from raw wafer maps for training the ensemble classifier. The ensemble decision tree model [25] and radon transform-based feature were used to identify wafer defects. Its final result is determined by the prediction results of multiple decision trees. Recently, there has also been also a method of wafer defect identification based on radon-based features and large-margin extreme learning machine [13], which achieved a high recognition accuracy on WM811K.

### 2.3. Wafer Defect Recognition Based on Deep Learning

Deep learning plays an important role in the field of visual image processing, and many researchers have applied it to wafer defect pattern recognition tasks. CNN-WDI [9] is a wafer defect automatic recognition model based on deep convolutional neural networks. It uses convolutional layers to extract valuable feature information from wafer images, adapting model regularization methods such as batch normalization and spatial dropout. CNN-WDI had defeated all previous machine-learning-based wafer defect recognition models. The performances of existing convolutional neural networks, such as ResNet, EfficientNetV2, ShuffleNetV2, MobileNetV2, and MobileNetV3, in wafer defect recognition tasks were analyzed [21]. The comparative experiments were conducted on the WM811K dataset, and the results showed that the average accuracy of each of these models was not significantly different, but MobileNetV3 had a faster inference speed than other models. Deformable convolutional units [14] were designed to selectively sample mixed-type wafer defects, which can extract high-quality feature information from wafer maps. MER-Net [18] was based on deformable convolution and Ghost convolution, and it performed with high accuracy for mixed-type wafer defect recognition. A dual-head convolutional neural network [12] trained via a semi-supervised learning method can effectively handle class-imbalanced classification of wafer defect patterns.

Adding attention mechanism to deep learning models can effectively improve the performance of wafer defect recognition. The SE (squeeze-and-excitation) module [26] and CBAM [27] are attention mechanism modules used to enhance convolutional neural networks. Models such as MobileNetV3 and EfficientNetV2 introduced an attention mechanism via the SE module. Adding CBAM to ResNet18 for wafer defect recognition [10] can improve accuracy, using a cosine normalization algorithm instead of fully connected layers to alleviate the problem of imbalanced data. Developing CBAM into a multi-branch attention module and integrating it into the ResNeXt50 backbone network [11] was undertaken to extract wafer defect features. The U-Net semantic segmentation model improved by CBAM and residual modules [28] achieved the effect of identifying mixed-type defects by training with only single-defect wafer maps. WM-PeleeNet [20], a lightweight wafer defect recognition model, was also improved by CBAM and it applied convolutional autoencoders to augment data.

Some researchers employed Transformer models with multi-head self-attention mechanisms for wafer defect recognition. The multi-head self-attention mechanism can encode the global contextual feature information of the wafer map and model the internal relationship between the wafer map and defect patterns. MSF-Trans [16] combined the convolutional attention mechanism and multi-head attention mechanism, integrating the advantages of the convolutional model innro a detailed feature learning and Transformer model in global feature learning, achieving high-accuracy mixed-type wafer defect recognition. The performances of four Transformer-based visual models, BEiT, FNet, ViT, and Swin Transformer, in wafer defect recognition tasks were explored in [15]. The comparative experiments were conducted on the Mixed-type WM38 dataset, and the results showed that the Swin Transformer had the highest average accuracy. Although Transformer models can extract the global feature information of images through the self-attention mechanism, their computational cost is high, and their inference speed is not as fast as convolutional models when the number of parameters is the same.

## 3. Proposed Method

To achieve high-accuracy and fast-inference for mixed-type wafer defect pattern recognition, this study designs a light-weight neural network model. The main steps are as follows:

1. First, enhance the attention mechanism of MBConv Block with CBAM and replace its activation function with ReLU, which is more suitable for wafer map data;
2. Second, redesign a separate downsampling layer to improve the inference speed; apply large kernel convolution to optimize the downsampling operations;
3. Third, build the light-weight neural network model based on the improved MBConv Block and large kernel convolution downsampling for mixed-type wafer defect recognition;
4. Fourth, introduce stochastic depth that can effectively alleviate the problem of overfitting in light-weight model training and improve the model’s generalization ability;
5. Finally, deploy the model as a TensorRT engine to improve the inference speed of the model on the GPU.

### 3.1. Proposed Model Architecture

**Figure 3:**
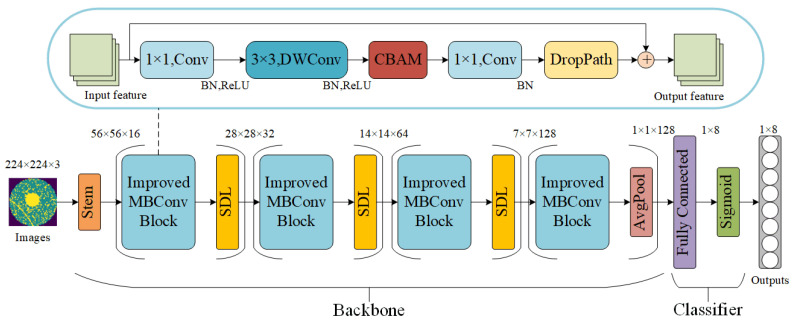
Proposed model architecture.

The lightweight model designed in this study is generally divided into two parts: a feature extraction backbone network and a classifier. The model architecture is shown in Figure 3.

The backbone network consists of four types of modules: Stem Block, Improved MBConv Block, SDL (Separate downsampling layer), and AvgPool. Stem Block is responsible for extracting basic features from input images and preparing the data for further processing. For Improved MBConv Block, the main feature extraction module of the model is an inverted residual structure with CBAM. This structure can enhance the performance of the model while effectively reducing the computational cost and the number of parameters. SDL is a downsampling block; it can effectively retain useful feature information while compressing the spatial resolution of feature maps. AvgPool aggregates the learned features and inputs them into the classifier.

The classifier consists of a fully connected layer and a Sigmoid function. The fully connected layer outputs eight values, each representing one of the eight types of wafer defects. These values are converted into probability values through the Sigmoid function. If the probability value output by the classifier is greater than or equal to 0.5, it is determined that the corresponding type of wafer defect exists.

### 3.2. The Parameters of Proposed Model

The parameters of deep neural networks are essential to the effectiveness of the network and the time required for generating results [29]. The detailed parameters of proposed model is shown in Table 1. The proposed model accepts 3 × 224 × 224 images as input and outputs 8 values. All the convolution layers and depthwise convolution layers are each followed by a batch normalization layer. All the batch normalization layers in Stem Blocks and SDL Blocks are followed by a ReLU, but only the first two batch normalization layers in IMBConv Blocks are followed by a ReLU. The Sigmoid function of the classifier is only activated during the model inference stage and not during the model training stage.

### 3.3. Improved MBConv Block

Improved MBConv Block is an improvement based on MBConv Block, which is the main block of MobileNetV3. MBConv Block starts with a 1 × 1 convolution for dimension expansion then applies a 3 × 3 depthwise convolution and a SE module for feature extraction, finally using a 1 × 1 convolution for dimension reduction. The residual connection is applied in MBConv Block.

The Improved MBConv Block replaces SE module with CBAM to further enhance the model’s feature extraction capability and incorporates DropPath block to implement stochastic depth techniques, which helps optimize model training. The structure of the Improved MBConv Block can be seen in Figure 3.

A 1 × 1 convolution, also known as pointwise convolution, is used for dimensionality expansion and reduction, adjusting the number of channels in the feature maps. A 1 × 1 convolution with input channels of $C_{in}$ and output channels of $C_{out}$ can be described as follows:

(1) $$output\left(C_{{out}_{j}}\right)=\sum_{i=1}^{C_{in}}weight\left(C_{{out}_{j}}\right)★input\left(C_{{in}_{i}}\right),$$

where ★ is the valid 2D cross-correlation operator, and weight is the convolutional kernel parameter with a size of 1 × 1 corresponding to the output channel.

Depthwise convolution extracts features expanded by the 1 × 1 convolution. Its calculation is only carried out within each channel. A 3 × 3 depthwise convolution with channels of *C* can be described as follows:

(2) $$output\left(C_{i}\right)=weight\left(C_{i}\right)★input\left(C_{i}\right),$$

where ★ is the valid 2D cross-correlation operator, and weight is the convolutional kernel parameter with a size of 3 × 3 corresponding to the channel.

In the Improved MBConv Block, the first 1 × 1 convolutional and the 3 × 3 depthwise convolutional are both followed by a batch normalization and a ReLU activation function, while the second 1 × 1 convolutional is only followed by a batch normalization. Batch normalization improves the gradient propagation, enhances the model stability, and has a certain effect of regularization. ReLU introduces nonlinear transformations, allowing the neural network to learn and represent more complex functional relationships.

#### 3.3.1. Convolutional Block Attention Module

**Figure 4:**
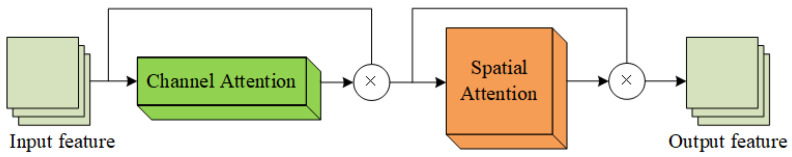
Convolutional block attention module.

Compared with the SE module, CBAM can better enhance the model’s attention to important feature information. Because CBAM combines channel attention and spatial attention; it can adaptively adjust the importance of features in both channel and spatial dimensions. But the SE module is just a specific implementation of channel attention. The structure of CBAM is shown in Figure 4.

**Figure 5:**
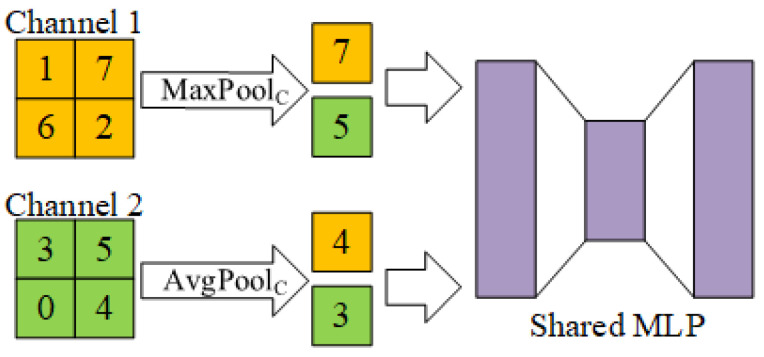
Channel attention.

The channel attention module performs global average pooling and global maximum pooling on the feature maps of each channel, obtaining the average and maximum features of the feature maps in each convolutional channel. These two features are fed into a weight sharing, two-layer fully connected neural network. During the model training process, this fully connected neural network will be trained as a channel weight. When the feature maps enter the channel attention module, the channel weight will adjust the feature response of each channel, thereby enhancing the feature expression ability of important channels. The channel attention operation is shown in Figure 5, and its calculation expression is as follows:

(3) $$W_{C}=Sigmoid\left(MLP\left({MaxPool}_{C}\left(input\right)\right)+MLP\left({AvgPool}_{C}\left(input\right)\right)\right),$$

(4) $$output=W_{C}\cdot input,$$

where $W_{C}$ is the channel weight.

**Figure 6:**
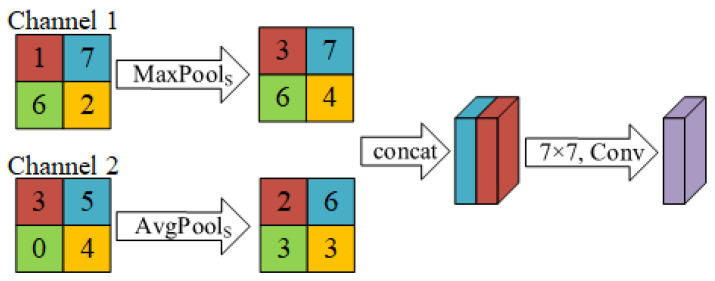
Spatial attention.

The spatial attention module calculates the average pooling feature map and maximum pooling feature map of all channels and obtains the average and maximum features of the feature map in space. These two features are concatenated and input into a 7 × 7 convolutional. During the model training process, this convolutional layer will be trained as a spatial weight. When the feature map enters the spatial attention module, the spatial weight will adjust the feature response of each pixel in the feature map, thereby enhancing the feature expression ability of important pixels. The spatial attention operation is shown in Figure 6, and its calculation expression is as follows:

(5) $$W_{S}=Sigmoid\left({Conv}_{7\times 7}\left(Concat\left({MaxPool}_{s}\left(input\right),{AvgPool}_{s}\left(input\right)\right)\right)\right),$$

(6) $$output=W_{S}\cdot input,$$

where $W_{S}$ is the spatial weight.

#### 3.3.2. Activation Function

ReLU is the most commonly used activation function in deep neural networks because of its advantages of simple calculation and easy differentiation. It can effectively alleviate the problem of gradient vanishing during model training. The calculation formula of ReLU is as follows:

(7) $$ReLU\left(x\right)=\left\{\begin{array}{cc} 0, & x\leq 0\\ x, & x>0 \end{array}\right..$$

HardSwish is used in the original MBConv Block, and it has a non-zero gradient when the input is negative, which can avoid the problem of neuron death and provide better performance in some cases. The calculation formula of HardSwish is as follows:

(8) $$HardSwish\left(x\right)=\left\{\begin{array}{cc} 0, & x\leq -3\\ x, & x\geq +3\\ x\cdot (x+3)/6, & otherwise \end{array}\right..$$

Whether to choose ReLU or HardSwish depends on the specific task and requirements. In our experiments, we found that ReLU performs better than HardSwish on wafer map data. The wafer map only has three categories of different pixels, representing background, normal circuits, and defective circuits. This type of image information is relatively simple, which makes neural networks more likely to overfit. ReLU activation function has the characteristic of sparsity, which enables neural networks to learn more sparse feature representations. This sparse representation can improve the model’s generalization and expression abilities. Moreover, the computation of ReLU is simpler than that of HardSwish, which gives it a faster inference speed. Therefore, replacing HardSwish in MBConv Block with ReLU is more suitable for wafer defect recognition tasks.

#### 3.3.3. Stochastic Depth

Stochastic Depth [30] is a regularization technique for improving deep learning model training. When the model is training, the DropPath Block will randomly drop layers in the network with a certain probability. During the forward inference in the model, the feature information from the previous layer is bypassed through residual connections, circumventing the dropped layers. During the backward propagation, the dropped layers are not involved in gradient computation and weight updates, thereby achieving a stochastic model depth. This approach can force neural networks to learn more diverse representations from different subsets of layers, reduce overfitting, and improve generalization ability, thereby enhancing the robustness of the model. The Pytorch-like pseudocode for DropPath is shown in Table 2. The probability of dropped layers increases with the depth of the model, as deeper layers are more challenging to train and optimize.

### 3.4. Large Kernel Convolution Downsampling

Downsampling is the process of selecting and compressing features. This operation helps reduce the number of model parameters, but it also leads to loss of feature information. Large kernel convolution has a larger receptive field so that it can capture a larger range of contextual information, which helps the model retain more important feature information during downsampling operations. Therefore, we use large kernel convolution with kernel size of 9 × 9 to optimize the downsampling operations.

#### 3.4.1. Stem Block

**Figure 7:**
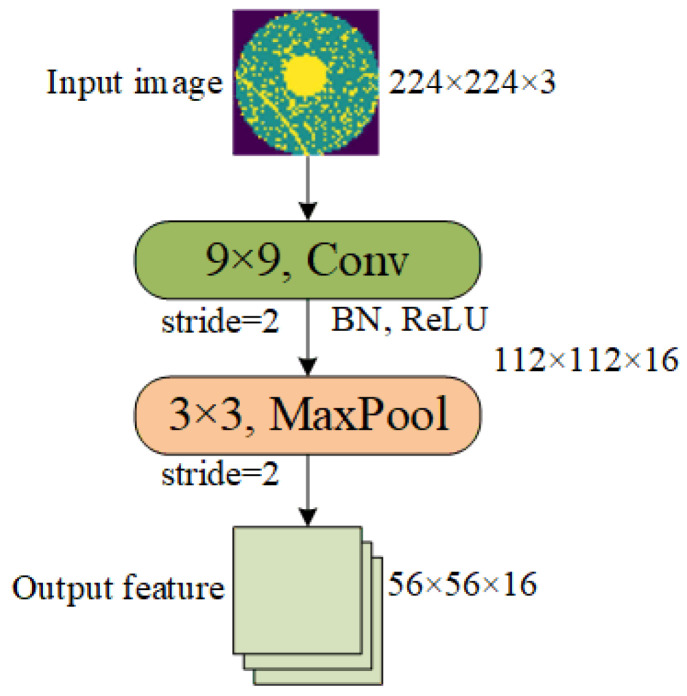
Stem Block.

The Stem Block of the proposed model is similar to ResNet’s, which consists of a 9 × 9 convolution with a stride of 2, a batch normalization, a ReLU, and a 3 × 3 max pooling with a stride of 2, as shown in Figure 7. The Stem block receives three-channel RGB images input with a resolution of 224 × 224. The convolution layer and the max pooling layer perform downsampling once each to extract and compress image features. Stem Block outputs sixteen-channel feature maps with a resolution of 56 × 56. This smaller size of feature maps is beneficial for improving the inference speed of the model.

#### 3.4.2. Separate Downsampling Layer

**Figure 8:**
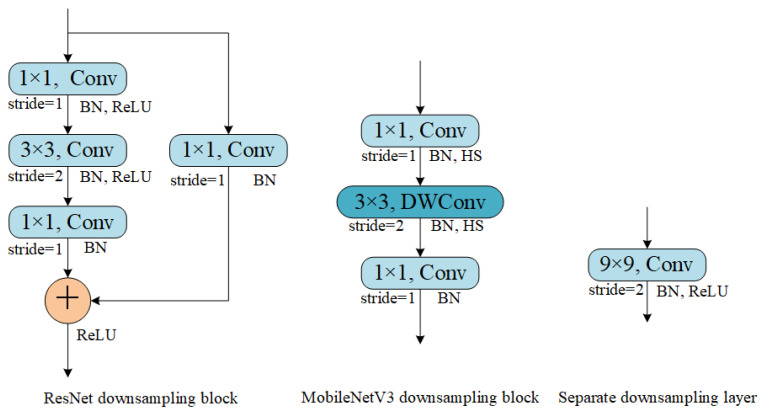
Comparison of different downsampling blocks.

Separate downsampling layer [31] is used for the spatial downsampling of feature maps and it consists only of a convolution and a batch normalization. SDL has the advantages of simple structure and fast inference speed, and its performance is not inferior to the traditional downsampling blocks such as ResNet’s and MobileNetV3’s. In this study, we apply 9 × 9 convolution to build the SDL and add ReLU to introduce more nonlinearity. The comparison of different downsampling blocks is shown in Figure 8.

### 3.5. TensorRT

TensorRT is a high-performance inference optimization library for deep learning networks. It optimizes the deep learning inference process by leveraging GPU architecture, memory management, and parallel computing techniques. The proposed model is deployed as a TensorRT engine after the model training, which helps accelerate the inference speed for wafer defect pattern recognition.

## 4. Experiments and Results

### 4.1. Data Preprocessing

The experiment of wafer defect pattern recognition is conducted on the Mixed-type WM38 dataset. There are 149 wafer maps in the category Near-Full, 866 wafer maps in the category Random, 2000 wafer maps in the category C+EL+S, and 1000 wafer maps in each of the other categories in the WM38 dataset. When the amount of data in some categories of the dataset is less than that of most other categories, it can lead to model bias in the learning and inference process, resulting in poor prediction performance for categories with less data. Therefore, the method of random flipping and random rotation is used to expand the number of data samples for Near-Full and Random categories to 1000.

The original wafer maps in the WM38 dataset are single-channel images with a resolution of 56 × 56. To obtain more detailed wafer defect information, the original wafer maps are adjusted to three-channel images with a resolution of 224 × 224. Normalizing the wafer map data can help the model better learn the features. The zero-mean unit-variance normalization method is used, and the calculation formula is as follows:

(9) x=(X−mean)/std,

where *X* is the original image, *x* is the normalized image, mean is the mean value of the wafer map data, and std is the standard deviation of the wafer map data.

The preprocessed wafer images are randomly shuffled to follow a uniform distribution and then divided into a training set, a validation set, and a test set in the ratio of 8:1:1. The total number of experimental wafer map data is 39,000, including 31,200 wafer maps for the training set, 3900 wafer maps for the validation set, and 3900 wafer maps for the test set. The model is trained on the training set, the hyperparameters are adjusted on the validation set, and the final performance is tested on the test set.

### 4.2. Experimental Settings and Evaluation Metrics

The experimental configuration is Intel Core i5-12400F CPU, 32 GB of RAM, Nvidia GeForce RTX4060 8G GPU, Windows 11, Python 3.10, CUDA 11.8, Pytorch 2.1, and TensorRT 8.6.1.

Each output value of the proposed model can represent whether the corresponding defect exists or not, which is obviously a multi-label classification task within the realm of binary classification. The BCEWithLogitsLoss is applicable to this task. It can convert the model output into predicted probability values through the sigmoid function and then use the probability values and the true label values (0 or 1) as inputs to calculate the binary cross-entropy loss for each binary classification problem. These losses are then summed to obtain the total loss. The BCEWithLogitsLoss function is used for model training, and its calculation formula is as follows:

(10) $$l\left(x,y\right)=L=\left\{l_{1},l_{2}\ldots ,l_{N}\right\},$$

(11) $$l_{n}=-\left[y_{n}\cdot log\sigma \left(x_{n}\right)+\left(1-y_{n}\right)\cdot log\left(1-\sigma \left(x_{n}\right)\right)\right],$$

where *x* is the predicted value of the model, *y* is the label, *N* is the batch size, log is the natural logarithm function, and σ is the Sigmoid function.

The model training utilizes an SGD (Stochastic gradient descent) optimizer with momentum; the update of model parameters can be written as:

(12) $$v_{t+1}=mv_{t}+g_{t+1},$$

(13) $$p_{t+1}=p_{t}-lrv_{t+1},$$

where lr is the learning rate, *m* is the momentum, *p* is the model parameters, *g* is the gradient, and *v* is the velocity. In the early stage of training, a higher learning rate can improve the convergence speed of the model. In the later stage of training, a lower learning rate is beneficial for the optimizer to further explore the optimal solution of the model, thereby improving model accuracy. Thus, we adopt a training strategy of learning rate decay. And the hyperparameter adjustments are made on the validation set. The initial learning rate for SGD is set to 0.02, and it decays to one-tenth of its current value after every 15 epochs of training. The momentum algorithm introduces an exponentially weighted average of historical gradients, making the parameter update direction more stable. The momentum parameter is set to 0.9. The number of epochs is set to 50, and the batch size is set to 64.

The evaluation of model performance adopts accuracy, precision, recall, and F1-score, which are commonly used evaluation metrics for deep learning visual classification model. The calculation formulae for these metrics are as follows:

(14) $$\mathrm{Accuracy}=\frac{TN+TP}{TP+FN+FP+TN},$$

(15) $$\mathrm{Precision}=\frac{TP}{TP+FP},$$

(16) $$\mathrm{Recall}=\frac{TP}{TP+FN},$$

(17) $$F1\mathrm{-score}=\frac{2\times \mathrm{Precision}\times \mathrm{Recall}}{\mathrm{Precision}\;+\;\mathrm{Recall}},$$

where TP is the true positive examples, FP is the false positive examples, FN is the false negative examples, and TN is the true negative examples.

In addition, the experiment also tested the model’s total parameters, FLOPs, and inference speed, which reflect the operational cost of the model.

### 4.3. Results and Analyses

**Figure 9:**
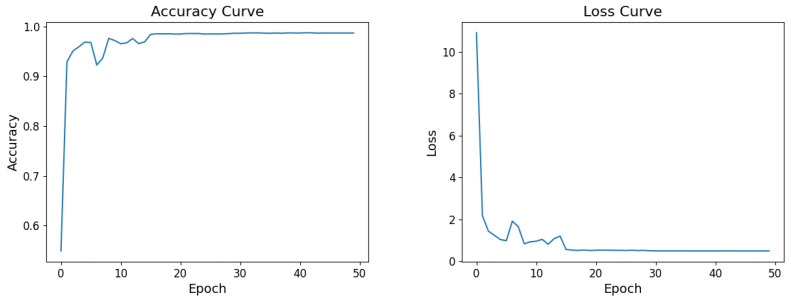
Accuracy curve and Loss curve.

The test set accuracy and loss changes of the proposed model during the learning process of 50 epochs are shown in Figure 9. The model parameters start to converge from the thirtieth epoch, and the accuracy of the model ultimately converges to 98.69%, with the loss ultimately converging to 0.4842.

The accuracy, precision, recall, and F1-score of the proposed model for each category are shown in Table 3. The proposed model has an accuracy of over 98% for most defects, over 95% for all defects, and an accuracy of 100% for 12 defects. The average accuracy of the model for a single defect is 99.15%, and the average accuracy for mixed-type defects exceeds 98%. The proposed model has an average accuracy of 98.69%, an average precision of 98.73%, an average recall of 98.74%, and an average F1-score of 98.73% on the test set, demonstrating excellent wafer defect recognition performance.

We replicated the wafer defect detection task on the WM38 dataset using some existing light-weight models and high-performance models and tested the relevant evaluation metrics. The proposed model outperforms other models in terms of accuracy, precision, recall, and F1-score, as shown in Table 4, the bold values represent the best results, and the following tables are the same.

The total parameters, FLOPs, and inference speed of each model are shown in Table 5. The proposed model has only 1.01M parameters and only 164.21M FLOPs, and it can process 317 images per second on RTX4060. Although the total parameters and FLOPs of the proposed model are similar to some existing light-weight models, it has the highest recognition accuracy and the fastest inference speed. The FLOPs of MobileNetV3_small is the lowest among all models. However, its inference speed is slower than our model. Because the structure of the propose model has a high degree of computational parallelism, resulting in faster inference speed on GPU, a hardware device for parallel computing. When the downsampling convolutions of the proposed model are set to 7 × 7 convolutions, the model has fewer parameters than other light-weight models, and the model accuracy is also higher than other models. But this leads to a slight decrease in performance, while the inference speed does not significantly improve. The proposed model achieves higher accuracy than the best-performing model among the existing ones, EfficientNetV2_s, while the inference speed is more than four times faster than it. When the model is deployed as a TensorRT engine, the experiment determines that it infers 1311 wafer maps per second on RTX4060.

The experimental results demonstrate that compared to existing models, the proposed model in this paper performs higher wafer defect recognition accuracy and faster inference speed, making it more effective in dealing with real wafer fabrication scenarios.

### 4.4. Ablation Experiments

Ablation experiments are used to evaluate the impact of different components on the overall performance of deep learning model. The results of the ablation experiments are shown in Table 6. The results show that CBAM can improve model performance, but it also increases computational complexity and leads to a decrease in inference speed. The inference speed of the model adapting ReLU activation function is faster than that adapting HardSwish, and the model performance is higher. The performance of the model with 9 × 9 large kernel convolution downsampling is significantly better than the commonly used 3 × 3 small kernel convolution. And stochastic depth can effectively improve model performance.

## 5. Conclusions

To achieve high accuracy and fast inference for mixed-type wafer defect pattern recognition, this study proposed a light-weight neural network model based on inverted residual convolution blocks with CBAM and large kernel convolutional downsampling layers. Experiments were conducted on the Mixed-type WM38 dataset, and the results showed that the proposed model achieved an accuracy of 98.69% with only 1.01M parameters. Compared with some popular existing models, such as ResNet50, MobileNetV3, EfficientNetV2_s, and SwinTransformer_t, the proposed model has higher recognition accuracy and higher inference speed. And the proposed model is 346% faster than the EfficientNetV2_s, with the highest recognition accuracy. Finally, we deployed the model as a TensorRT engine and found that it can process 1311 wafer maps per second, which means that the model is further accelerated by 314%. Thus, this study has improved the efficiency of mixed-type wafer defect pattern recognition.

There are also some limitations to our work. In this article, to address the issue of imbalanced data categories, we employed the methods of flipping and rotation for data augmentation. Although these methods are simple and convenient, the obtained data do not possess diverse feature information, which does not help to improve the generalization ability of the model. Moreover, TensorRT acceleration is a library based on Nvidia GPU and cannot be depolyed on other devices.

In our future work, we plan to generate some wafer maps through generative deep learning models such as VAE or GAN to help the model learn more complex and diverse wafer defect information and improve its generalization ability. Meanwhile, we also plan to introduce more advanced deep learning techniques to further optimize the model and improve its structural parallelism to fully utilize the parallel computing capabilities of the GPU.

## Figures and Tables

**Table 1:** The detailed parameters of proposed model.

Block	Residual	Sublayers	Stride	Padding	Output Size
Stem	×	9 × 9 conv, 3 × 3 maxpool	2, 2	4, 1	16 × 56 × 56
IMBConv	√	1 × 1 conv, 3 × 3 dwconv, 1 × 1 conv	1, 1, 1	0, 1, 0	16 × 56 × 56
SDL	×	9 × 9 conv	2	4	32 × 28 × 28
IMBConv	√	1 × 1 conv, 3 × 3 dwconv, 1 × 1 conv	1, 1, 1	0, 1, 0	32 × 28 × 28
SDL	×	9 × 9 conv	2	4	64 × 14 × 14
IMBConv	√	1 × 1 conv, 3 × 3 dwconv, 1 × 1 conv	1, 1, 1	0, 1, 0	64 × 14 × 14
SDL	×	9 × 9 conv	2	4	128 × 7 × 7
IMBConv	√	1 × 1 conv, 3 × 3 dwconv, 1 × 1 conv	1, 1, 1	0, 1, 0	128 × 7 × 7
AvgPool	×	avgpool	-	-	128 × 1 × 1
Classifier	×	fc(128, 8), sigmoid	-	-	8

**Table 2:** The PyTorch-like pseudocode for DropPath. (# indicates note, * indicates multiplication.)

# x – input feature maps with [N, C, W, H] shapes
# drop_prob – the probability of layers to be dropped

keep_prob = 1 − drop_prob

# generate drop matrix
shape = (x.shape[0],) + (1,) * (x.ndim − 1)
random_tensor = keep_prob + torch.rand(shape, dtype = x.dtype, device = x.device)
random_tensor.floor_()

# regularize and execute drop
output = x.div(keep_prob) * random_tensor

**Table 3:** The accuracy, precision, recall, and F1-score of the proposed model (%).

Defect	Accuracy	Precision	Recall	F1-Score
Normal	100.00	100.00	97.94	98.96
Center	98.21	98.21	98.21	98.21
Donut	100.00	100.00	100.00	100.00
Edge_Loc	100.00	100.00	96.34	98.14
Edge_Ring	98.95	98.95	97.92	98.43
Loc	98.11	98.11	100.00	99.05
Near_Full	100.00	100.00	99.09	99.54
Scratch	99.05	99.05	100.00	99.52
Random	98.84	98.84	100.00	99.42
C+EL	98.99	98.99	98.00	98.49
C+ER	100.00	100.00	100.00	100.00
C+L	98.02	98.02	98.02	98.02
C+S	100.00	100.00	96.77	98.36
D+EL	99.07	100.00	98.15	99.07
D+ER	97.12	97.12	97.12	97.12
D+L	100.00	100.00	99.13	99.56
D+S	97.92	97.92	98.95	98.43
EL+L	97.96	97.96	100.00	98.97
EL+S	100.00	100.00	100.00	100.00
ER+L	97.14	97.14	100.00	98.55
ER+S	99.01	99.01	100.00	99.50
L+S	100.00	100.00	97.20	98.58
C+EL+L	97.59	97.59	98.78	98.18
C+EL+S	99.49	99.49	100.00	99.75
C+ER+L	99.07	100.00	99.07	99.53
C+ER+S	100.00	100.00	99.03	99.51
C+L+S	96.12	96.12	99.00	97.54
D+EL+L	95.79	95.79	100.00	97.85
D+EL+S	96.75	96.75	98.35	97.54
D+ER+L	96.84	96.84	98.92	97.87
D+ER+S	100.00	100.00	93.39	96.58
D+L+S	98.08	98.08	100.00	99.03
EL+L+S	97.83	97.83	100.00	98.90
ER+L+S	100.00	100.00	98.13	99.06
C+L+EL+S	98.91	98.91	98.91	98.91
C+L+ER+S	99.01	99.01	100.00	99.50
D+L+EL+S	98.90	98.90	96.77	97.83
D+L+ER+S	97.25	97.25	99.07	98.15
Single defect	99.15	99.15	98.95	99.04
Tow defects	98.86	98.94	98.72	98.82
Three defects	98.13	98.21	98.72	98.45
Four defects	98.52	98.52	98.69	98.60
Average	98.69	98.73	98.74	98.73

**Table 4:** The accuracy, precision, recall, and F1-score of each model (%).

Model	Accuracy	Precision	Recall	F1-Score
MobileNetV2	97.56	97.57	97.64	97.58
MobileNetV3_small	97.41	97.48	97.51	97.48
MobileNetV3_large	97.82	97.93	97.99	97.94
ShuffleNetV2_x1.0	95.44	95.49	96.60	95.51
MobileViT_XXS	97.87	97.83	97.85	97.82
PeleeNet	96.41	96.52	96.60	96.51
ResNet50	96.92	97.32	97.38	97.31
EfficientNetV2_s	98.33	98.36	98.43	98.38
SwinTransformer_t	97.41	97.44	97.49	97.46
Proposed model	**98.69**	**98.73**	**98.74**	**98.73**

**Table 5:** The total parameters, FLOPs, and inference speed of each model.

Model	Total Parameters (M)	FLOPs (M)	Images/s	Accuracy (%)
MobileNetV2	2.23	326.22	247	97.56
MobileNetV3_small	1.53	**61.46**	260	97.41
MobileNetV3_large	4.21	233.57	220	97.82
ShuffleNetV2_x1.0	1.26	151.69	231	95.44
MobileViT_XXS	0.95	273.35	143	97.87
PeleeNet	1.65	516.73	131	96.41
ResNet50	23.52	4131.71	192	96.92
EfficientNetV2_s	20.19	2900.67	71	98.33
SwinTransformer_t	18.86	2977.34	99	97.41
Proposed model (7 × 7)	**0.67**	106.41	323	98.46
Proposed model	1.01	164.21	317	**98.69**
Proposed model (TRT)	1.01	164.21	**1311**	**98.69**

**Table 6:** The results of the ablation experiments.

Attention Block	Activation Function	Stochastic Depth	Downsample Kernel Size	Accuracy (%)	Images/s
SE	Hardswish	×	3 × 3	97.38	**427**
CBAM	Hardswish	√	3 × 3	97.79	310
CBAM	Hardswish	√	7 × 7	98.41	305
CBAM	ReLU	√	7 × 7	98.46	323
SE	ReLU	×	9 × 9	97.82	425
SE	ReLU	√	9 × 9	98.03	411
CBAM	ReLU	×	9 × 9	98.49	322
CBAM	Hardswish	√	9 × 9	98.54	276
CBAM	ReLU	√	9 × 9	**98.69**	317

## Data Availability

The raw data supporting the conclusions of this article will be made available by the authors on request.
